# A cross-sectional study on illness perception and resiliency among cancer patients, in Bhaktapur cancer hospital

**DOI:** 10.1371/journal.pone.0356524

**Published:** 2026-09-17

**Authors:** Pooja Panth, Abaru Panta, Kabita Raj Cadel, Prajwol Baniya

**Affiliations:** 1 Department of Psychology, Padmakanya Campus, Kathmandu, Nepal; 2 Department of Health Care Administration, St. Lawrence College, Kingston, Canada; Sunrise University, INDIA

## Abstract

Cancer, characterized by uncontrollable abnormal cell growth, is one of the leading causes of death worldwide. According to the WHO, cancer accounted for nearly 1 in 6 deaths in 2020. Most research has focused on the physical well-being and quality of life of cancer patients. However, with increasing emphasis on psychological aspects, particularly illness perception and resilience, a cross-sectional hospital-based study was conducted to assess the impact of illness perception on the resiliency of cancer patients. This correlational quantitative study used convenient non-probability sampling at Bhaktapur Cancer Hospital, a national-level cancer center, enrolling a total of 170 participants. The Brief Illness Perception Questionnaire (IPQ-B) and the Connor-Davidson Resilience Scale (CD-RISC 10) were used to assess illness perception and resiliency, respectively. More than half of the respondents (57.6%) reported experiencing a high level of perceived threat from their illness. While 75.9% of participants showed low level of resilience. A significant modest negative correlation was found between illness perception (BIPQ) and resilience (CDRISC) (−0.177,p < 0.05). No significant differences in resilience or illness perception were found based on gender or socioeconomic status. However, a significant difference in resilience was observed based on educational background (p = 0.033), while illness perception did not vary significantly with education. These findings suggest that addressing illness perception through targeted psychoeducation and supportive therapeutic approaches may enhance resilience among cancer patients and improve their quality of life.

## Introduction

When abnormal cell growth becomes uncontrolled, it can develop into cancer, affecting any organ or tissue in the body. Also known as a neoplasm or malignant tumor, cancer results from DNA mutations that may occur spontaneously or be inherited. It encompasses a group of diseases marked by uncontrolled cell proliferation with the potential to invade or spread to other areas [1]. Globally, cancer remains one of the foremost causes of mortality, accounting for approximately one in six deaths in 2020 (WHO). More than 120 types have been identified, typically named after the organ or tissue from which they originate. Common categories include carcinoma (affecting the skin or organ linings), sarcoma (in connective tissues), leukemia (in the bone marrow), and lymphoma or myeloma (in the immune system). In 2020, about 18.1 million new cases were reported worldwide, with higher rates in men than women. Breast cancer was the most frequently diagnosed (11.7%), followed by lung, colorectal, prostate, and stomach cancers [2]. Lung cancer accounted for the highest number of deaths (18%), followed by colorectal, liver, stomach, and breast cancers. Contributing risk factors include tobacco and alcohol use, environmental pollution, viral infections such as HIV, HPV, Hepatitis B and C, and unhealthy dietary patterns [3]. In Nepal, 20,508 new cases were recorded in 2020, with incidence rates of 41.4 per 100,000 among females and 38.5 among males. Lung cancer was particularly prevalent, attributed to high smoking rates among Nepalese women [4]. Treatments for cancer generally involves surgery, radiation, chemotherapy, immunotherapy, or hormonal therapy, with the choice depending on individual and tumor-related factors. Chemotherapy, which employs antineoplastic agents, targets rapidly dividing cells and acts systemically to control disease progression [5]. Beyond its physical burden, a cancer diagnosis deeply impacts the emotional and psychological well-being of patients and their families. Feelings of anxiety, sadness, and distress are common, and many survivors continue to struggle with mental health challenges even after treatment. Illness perception, the way individuals cognitively and emotionally understand their condition, plays a key role in how they cope and respond to treatment [6]. Such perceptions shape personal interpretations of the illness and influence health behaviors and recovery [7].

Resilience, on the other hand, refers to the ability to adapt effectively in the face of stress or adversity [8]. According to the American Psychological Association (2010), it is the process of maintaining psychological stability despite hardship. Research shows that individuals with strong resilience display better acceptance, adaptive coping, improved quality of life, and emotional balance [9,10]. Depression affects between 20–50% of cancer patients and may coexist with adjustment disorders or PTSD [11]. Despite advances in medical treatment, many patients continue to face significant emotional and psychological struggles. Illness perception greatly influences how patients manage and adapt to their disease; however, there is limited evidence linking it to resilience, particularly in relation to illness severity. This study aims to explore how perceptions of illness affect resilience among cancer patients while considering disease progression. Cancer impacts not only the body but also the psychological, social, and behavioral dimensions of life. Many patients experience emotional distress, sleep disturbances, and decreased quality of life [12]. Understanding how individuals interpret and cope with their diagnosis is vital for improving mental health outcomes. Studies indicate that factors such as illness perception, age, education, and socioeconomic background play an important role in shaping resilience [13]. In Nepal, where cancer is still viewed as a financially and emotionally overwhelming condition, these psychosocial aspects often receive little attention. This research therefore seeks to highlight the relationship between illness perception and resilience among Nepalese cancer patients, providing insights that could guide mental health interventions and policy development within cancer care settings.

## Materials and methods

A hospital-based cross-sectional descriptive study using a quantitative approach was conducted at Bhaktapur Cancer Hospital, a 150-bedded national-level hospital located in Dudhpati, Bhaktapur District, Bagmati Province. carried out over six months, from January 2023 to August 202 The study was conducted among 170 participants. Participants were selected based on convenient non-probability sampling. Adult patients (aged 20–60 years) diagnosed with any type or stage of cancer and attending OPD or admitted to IPD were included in the study, while those unable to respond due to poor health and those who had already recovered from cancer were excluded. The study was carried out over six months from January 2023 to August 2023.

Data were collected individually through structured questionnaires. Three standardized tools were used. The Illness Perception Questionnaire–Brief (IPQ-B) is a nine-item scale measuring cognitive and emotional representations of illness on a 10-point Likert scale [14]. The causal representation item was excluded as it was not relevant to the study objectives. Scores were categorized into low (0–44), moderate (45–49), and high (50–80) levels of illness perception. The Connor-Davidson Resilience Scale (CD-RISC-10) was used to assess flexibility, self-efficacy, emotion regulation, and optimism through 10 items rated on a 5-point Likert scale (0–4), with higher scores indicating greater resilience. The tool shows good internal consistency (α = 0.88–0.89) and test-retest reliability [15]. The Modified Kuppuswamy Scale was used to assess socioeconomic status based on education, occupation, and income, adapted for the Nepalese context [16].

The tools were pretested on 10% of the total sample to ensure clarity and reliability. Necessary modifications were made based on feedback. Validity was maintained through an extensive literature review, use of standardized tools, and expert review during proposal development. Reliability was further ensured through pretesting and consistent data collection by the researcher. After data collection, all responses were cleaned, edited, and entered into Microsoft Excel, and the dataset was then exported to SPSS version 25 for statistical analysis. Descriptive statistics, including frequencies and percentages, were used to summarize demographic and clinical data, while inferential analyses such as correlation, independent t-tests, and one-way ANOVA were performed to examine the association between illness perception, resilience, and illness severity.

Ethical approval for the study was obtained from Padmakanya Campus, the Nepal Health Research Council (NHRC), and Bhaktapur Cancer Hospital. Written and verbal informed consent was obtained from all participants. Confidentiality and privacy were maintained throughout the study, and all ethical principles were followed to ensure participants’ safety, dignity, and autonomy.

## Results and discussion

Table 1 indicates the descriptive demographic information of participants. Among the total sample size of 170, majority of respondent were male, i.e., 51.8% and minority were female, i.e., 48.2%. Based on age, highest number of respondents belonged to the age group of 50–59 which was 22.9% The mean age of the respondents was 50.

**Table 1:** Socio Demographic and Socio-Economic Characteristics of the respondents (n = 170).

Variables		Frequency (n)	Percent (%)
Sex	Male Female	88 82	51.8 48.2
Ethnicity	Brahmin Chhetri Newar Tamang Magar Rai Gurung Others	49 43 23 17 14 5 3 16	28.8 25.5 13.5 10.0 8.2 2.9 1.8 9.4
Religion	Hindu Buddhists Christians	147 14 9	86.47 8.2 5.29
Marital Status	Married Unmarried	158 12	92.9 7.1
Education Level	Illiterate Non-Formal Study Primary Secondary Higher secondary Bachelor Master’s and above	58 2 8 32 25 29 16	34.11 1.17 4.70 18.8 14.70 17.05 9.41
Socio-economic Status	Lower middle class Upper Middle class Upper lower class Upper class	54 52 42 22	31.76 30.5 24.7 12.94

Similarly, the highest proportion of participants belonged to Brahmin Caste (28.8%) followed by Chhetri (25.3%), Newar (13.5%), and Others In the study, majority of the respondents were Hindu (86.5%) followed by Buddhist (8.2%) and Christian (5.29%) and92.9% of respondents were married, and 7.1% of respondents were unmarried. Less than half of the respondents (34.1%) were illiterate followed by Non-formal study (1.2%), Primary level (4.7%) and Secondary level (18.8%). Socio-economic characteristics was analysed by using Kuppuswamy scale and most of the respondents were in the lower middle class (37.76%) followed by upper middle class (30.5%), upper lower class (24.7%) and upper class (12.94%).

Among the study population, 21 different types of cancer were found based on the organ distribution of Cancer. Majority of the respondents were suffering from Breast cancer (14.1%) followed by Uterine cancer (12.4%), Intestine cancer (11.2%), Lung’s cancer (11.2%), Prostate cancer (7.1).

Table 2 presents the Pearson correlation analysis between the Connor–Davidson Resilience Scale (CD-RISC) scores and the Brief Illness Perception Questionnaire (BIPQ) scores. The results revealed a weak but statistically significant negative correlation between resilience and illness perception (*r* = −0.177, *p* < .05). This finding indicates that participants with higher resilience tended to report lower illness perception scores. Although the correlation was statistically significant, its magnitude was weak, suggesting that resilience was only modestly associated with illness perception in the study population.

**Table 2:** Co-relation between CDRISC and BIPQ.

Variables	CDRISC Score (Pearson correlation coefficient)
**BIPQ scale score**	−0.177*

*p≤.05, n=170

An independent-samples t test was conducted to compare resilience (CD-RISC) and illness perception (BIPQ) scores between male and female participants (Table 3). There was no statistically significant difference in resilience scores between males (M = 23.64, SD = 7.58) and females (M = 22.70, SD = 7.37), t(168) = 0.82, p = .908. Although males demonstrated a slightly higher mean resilience score than females (mean difference = 0.94), the difference was not statistically significant. Similarly, there was no statistically significant difference in illness perception (BIPQ) scores between males (M = 49.32, SD = 13.94) and females (M = 50.41, SD = 12.01), t(168) = −0.55, p = .115. Females had a marginally higher mean illness perception score than males; however, this difference was also not statistically significant.

**Table 3:** Independent sample t-test based on Sex.

Scale	Male (*n* = 88), *M* (SD)	Female (*n* = 82), *M* (SD)	Mean Difference	*t*(168)	*p*
CD-RISC	23.64 (7.58)	22.70 (7.37)	0.94	0.82	.908
BIPQ	49.32 (13.94)	50.41 (12.01)	−1.09	−0.55	.115

A one-way ANOVA was conducted to examine differences in resilience (CD-RISC) and illness perception (BIPQ) according to education level and socio-economic status among 170 participants (Table 4). Education level was significantly associated with resilience scores, F(5, 164) = 2.73, *p* = .021, indicating that resilience differed across educational groups. Participants with primary education had the highest resilience scores (*M* = 28.88, *SD* = 6.51), whereas illiterate participants had the lowest scores (*M* = 21.07, *SD* = 7.01). In contrast, education level was not significantly associated with illness perception, F(6, 163) = 0.44, *p* = .854. Similarly, socio-economic status was not significantly associated with resilience, F(3, 166) = 1.92, *p* = .128, or illness perception, F(3, 166) = 0.60, *p* = .615.

**Table 4:** One-way ANOVA test based on education and socio-economic status.

Variable	Category	*n*	CD-RISC *M* (SD)	F	*p*	BIPQ *M* (SD)	F	*p*
**Education**	Illiterate	58	21.07 (7.01)	2.73	.021	49.10 (12.42)	0.44	.854
	Non-formal study	2	27.00 (11.31)			52.00 (9.89)		
	Primary level	8	28.88 (6.51)			43.75 (9.00)		
	Secondary level	32	22.41 (7.98)			50.25 (13.48)		
	Higher secondary	25	23.76 (8.34)			51.24 (14.55)		
	Bachelor’s	29	25.62 (6.06)			51.28 (13.23)		
	Master’s and above	16	23.75 (7.20)			49.75 (15.96)		
**Socio-economic Status**	Upper lower	42	21.40 (7.31)	1.92	.128	48.45 (11.13)	0.60	.615
	Lower middle	54	22.54 (7.58)			51.54 (11.97)		
	Upper middle	52	24.65 (7.52)			49.98 (13.65)		
	Upper class	22	24.68 (6.90)			48.05 (17.11)		

The present study examined illness perception and resilience among cancer patients. The findings revealed a weak but statistically significant negative correlation between resilience and illness perception, indicating that participants with higher resilience tended to perceive their illness as less threatening. This finding is consistent with the study by Chow (2021), which also reported a negative association between resilience and illness perception among cancer patients. No significant differences were found in resilience or illness perception according to sex. Although males had slightly higher resilience scores and females reported slightly higher illness perception scores, these differences were not statistically significant. This finding differs from that of Portnoy et al. (2018), who reported higher resilience among males in patients experiencing chronic illness and traumatic events.

Education level was significantly associated with resilience, with participants having higher educational attainment demonstrating greater resilience. This finding is consistent with Bonanno et al. (2006), who also reported that higher education was associated with greater resilience. However, education level was not significantly associated with illness perception. Similarly, socio-economic status was not significantly associated with either resilience or illness perception. This finding contrasts with the study by Fesesterling et al. (2022), which found that resilience was positively associated with higher education and better income.

## Conclusion

Thus, the findings suggest that resilience plays an important role in shaping how cancer patients perceive their illness. Strengthening resilience through psychosocial support, patient education, and counseling may help reduce negative illness perceptions and improve psychological well-being. Further studies with larger sample sizes are recommended to better understand the factors influencing resilience and illness perception among cancer patients in Nepal.

### Limitation

This study has several limitations as a cross-sectional design, which limits the ability to establish causal relationships between illness perception and resilience, sampling technique which may limit the generalizability of the findings to all cancer patients in Nepal. Similarly, the study was relied on self-reported questionnaires, which could cause recall and social desirability bias. The unequal distribution of participants across educational categories and different cancer types may have influenced the comparison of resilience and illness perception among subgroups.

### Recommendation

Future studies should include larger and more representative samples from multiple healthcare institutions across Nepal to improve the generalizability of the findings. Longitudinal studies could be done to understand changes in illness perception and resilience over the course of cancer diagnosis and treatment. Other psychosocial factors may be included in study to explain resilience. The study also shows need of intervention studies evaluating the effectiveness of psychoeducation, counseling and resilience-building programs among cancer patients to improve psychological well-being and treatment outcomes.

## Supporting information

S1 Data Revised Dataset. (XLSX)

## References

[1] Guduru H, Jeevanagi SKR, Nigudgi S, Bhandare SV. A prospective study on the prescription pattern of anti-cancer drugs and adverse drug reaction in a tertiary care hospital. Int J Basic Clin Pharmacol. 2019;8(2):200. doi: 10.18203/2319-2003.ijbcp20190134

[2] Siegel RL, Miller KD, Fuchs HE, Jemal A. Cancer statistics, 2022. CA Cancer J Clin. 2022;72:7–33. 10.3322/caac.21708 35020204

[3] Shrestha G, Thakur RK, Singh R, Mulmi R, Shrestha A, Pradhan PMS. Cancer burden in Nepal, 1990–2017: an analysis of the Global Burden of Disease study. PLoS One. 2021;16:1–10. 10.1371/journal.pone.0255499 PMC8330909 34343216

[4] Poudel KK, Huang Z, Neupane PR, Steel R. Prediction of the Cancer Incidence in Nepal. Asian Pac J Cancer Prev. 2017;18(1):165–8. doi: 10.22034/APJCP.2017.18.1.165 28240512 PMC5563094

[5] Bepari A, Sakre N, Rahman I, Niazi SK, Dervesh AM. Assessment of drug utilization of anticancer drugs using WHO prescribing indicators in a government tertiary care hospital of the Hyderabad-Karnataka region of India. Open Access Maced J Med Sci. 2018;6:394–424. 10.3889/oamjms.2019.249 PMC6490503 31049108

[6] Broadbent E, Wilkes C, Koschwanez H, Weinman J, Norton S, Petrie KJ. A systematic review and meta-analysis of the Brief Illness Perception Questionnaire. Psychol Health. 2015;30(11):1361–85. doi: 10.1080/08870446.2015.1070851 26181764

[7] Al-Smadi AM, Ashour A, Hweidi I, Gharaibeh B, Fitzsimons D. Illness perception in patients with coronary artery disease: A systematic review. Int J Nurs Pract. 2016;22(6):633–48. doi: 10.1111/ijn.12494 27687787

[8] Luthar SS, Cicchetti D, Becker B. The construct of resilience: a critical evaluation and guidelines for future work. Child Dev. 2000;71(3):543–62. doi: 10.1111/1467-8624.00164 10953923 PMC1885202

[9] Bonanno GA. Loss, trauma, and human resilience. Am Psychol. 2004;59:20–8. 10.1037/0003-066X.59.1.20 14736317

[10] Bonanno GA, Galea S, Bucciarelli A, Vlahov D. Psychological resilience after disaster: New York City in the aftermath of the September 11th terrorist attack. Psychol Sci. 2006;17(3):181–6. doi: 10.1111/j.1467-9280.2006.01682.x 16507055

[11] Gregurek R, Bras M, Dordević V, Ratković A-S, Brajković L. Psychological problems of patients with cancer. Psychiatr Danub. 2010;22(2):227–30. 20562751

[12] Seiler A, Jenewein J. Resilience in Cancer Patients. Front Psychiatry. 2019;10:208. doi: 10.3389/fpsyt.2019.00208 31024362 PMC6460045

[13] Chiu H-C, Lin C-Y, Kuo Y-L, Hou W-L, Shu B-C. Resilience among women with breast cancer surviving longer than five years: The relationship with illness perception and body image. Eur J Oncol Nurs. 2023;62:102254. doi: 10.1016/j.ejon.2022.102254 36621263

[14] Broadbent E, Petrie KJ, Main J, Weinman J. The brief illness perception questionnaire. J Psychosom Res. 2006;60(6):631–7. doi: 10.1016/j.jpsychores.2005.10.020 16731240

[15] Connor KM, Davidson JRT. Development of a new resilience scale: the Connor-Davidson Resilience Scale (CD-RISC). Depress Anxiety. 2003;18(2):76–82. doi: 10.1002/da.10113 12964174

[16] Kuppuswamy B. Manual of Socioeconomic Status (Urban). Delhi: Manasayan; 1981.

